# The Warburg effect: a score for many instruments in the concert of cancer and cancer niche cells

**DOI:** 10.1007/s43440-023-00504-1

**Published:** 2023-06-19

**Authors:** Martyna Jaworska, Julia Szczudło, Adrian Pietrzyk, Jay Shah, Sonia E. Trojan, Barbara Ostrowska, Kinga A. Kocemba-Pilarczyk

**Affiliations:** 1 Chair of Medical Biochemistry, Faculty of Medicine, Jagiellonian University Medical College, Kraków, Poland; 2 Faculty of Chemistry, Jagiellonian University, Kraków, Poland; 3 Government Medical College Miraj, Miraj, Maharashtra India

**Keywords:** Warburg effect, Tumor metabolism, Aerobic glycolysis, Metabolic adaptation of cancer cells, Reverse Warburg effect

## Abstract

Although Warburg's discovery of intensive glucose uptake by tumors, followed by lactate fermentation in oxygen presence of oxygen was made a century ago, it is still an area of intense research and development of new hypotheses that, layer by layer, unravel the complexities of neoplastic transformation. This seemingly simple metabolic reprogramming of cancer cells reveals an intriguing, multi-faceted nature that may link various phenomena including cell signaling, cell proliferation, ROS generation, energy supply, macromolecules synthesis/biosynthetic precursor supply, immunosuppression, or cooperation of cancerous cells with cancer-associated fibroblasts (CAFs), known as reversed Warburg effect. According to the current perception of the causes and consequences of the Warburg effect, PI3K/Akt/mTOR are the main signaling pathways that, in concert with the transcription factors HIF-1, p53, and c-Myc, modulate the activity/expression of key regulatory enzymes, including PKM2, and PDK1 to tune in the most optimal metabolic setting for the cancer cell. This in turn secures adequate levels of biosynthetic precursors, NADPH, NAD^+^, and rapid ATP production to meet the increased demands of intensively proliferating tumor cells. The end-product of “aerobic glycolysis”, lactate, an oncometabolite, may provide fuel to neighboring cancer cells, and facilitate metastasis and immunosuppression together enabling cancer progression. The importance and possible applicability of the presented issue are best illustrated by numerous trials with various agents targeting the Warburg effect, constituting a promising strategy in future anti-cancer regimens. In this review, we present the key aspects of this multifactorial phenomenon, depicting the mechanisms and benefits behind the Warburg effect, and also pointing to selected aspects in the field of anticancer therapy.

## Introduction

Cancer cells arise as a result of mutations that occur in the genome of somatic cells leading to their clonal proliferation in a self-sustaining manner. Mutations lead to the formation of a new genotype, which directly shapes the cellular phenotype of tumors, which in turn differs significantly from that of normal cells. These differences are readily apparent at the level of the basal metabolism involved in energy and biomass production [1]. In normal cells, glucose is taken up by specific transporters (GLUTs—glucose transporters) and subsequently metabolized mainly via the glycolysis pathway, converting one molecule of glucose to two molecules of pyruvate, accompanied by NAD^+^ (Nicotinamide adenine dinucleotide, oxidized form) reduction. Assuming sufficient oxygen supply, pyruvate is then oxidized and NAD^+^, necessary for glycolysis, is recovered by the action of NADH (Nicotinamide adenine dinucleotide, reduced form) shuttles (translocating electrons from cytosolic to a mitochondrial pool of reducing equivalents) and the electron transport chain. It is now a well-known fact that cancer cells have a greater predisposition for lactic acid formation even under adequate oxygen supply, referred to as aerobic glycolysis, which is rather unusual for normal cells [1]. Exceptions to this dictum are mammalian cells undergoing intense proliferation, such as pluripotent stem cells, cells of the immune system, and endothelial cells, as well as regenerating tissues. For these cell types, such kind of metabolic shift was also confirmed and noted as a “hallmark of rapid proliferation” [2]. This seemingly simple metabolic change, first observed by Otto Warburg, entails a still-to-be-explored network of intra- and intercellular interactions involved in fine-tuning tumor metabolism to promote its progression. Therefore, this review aims to show the multifaceted nature of the Warburg effect, emphasizing its potential importance for the development of new therapeutic regimens enabling effective, specific, and safe treatment of cancer.

## Warburg effect—a still unresolved issue

### Direct effect on transformed cells

**Fig. 1:**
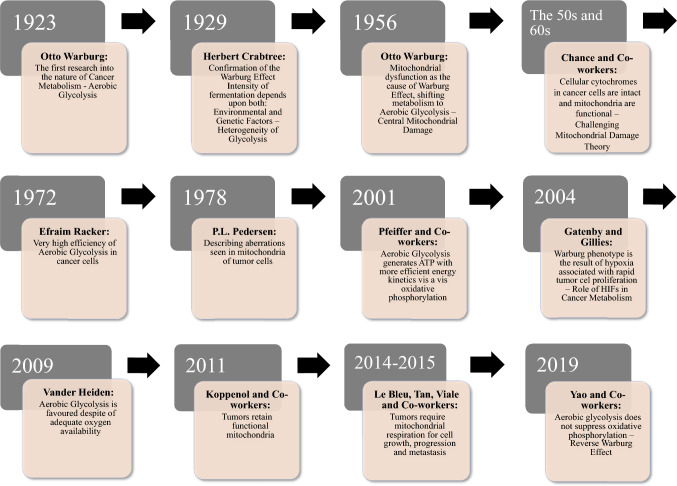
Major discoveries related to Warburgs’ original thought evolution [8, 11, 137]

A century ago, Otto Warburg working with a rat seminal vesicle tumor unexpectedly observed a lower level of oxygen consumption by rapidly proliferating cells accompanied by high glucose uptake and lactate formation [3]. Experiments performed in various conditions in terms of glucose and/or oxygen supply led him to conclude that besides increased glucose consumption and lactate production under normal oxygen supply, oxidative metabolism is also required for cancer cell survival [4, 5]. Eventually, in contradiction to their own observations, Warburg claimed mitochondria damage as a reason for cancer transformation [6, 7]. Since then, scientists have repeatedly returned to the subject of aerobic glycolysis, named after the discoverer, the Warburg effect [8], and the last 10 years have seen a renewed interest and increasingly vigorous research into cancer cell metabolism. The aim was not only to understand the basics governing the Warburg effect, but also to comprehend the purpose of such a metabolic pathway chosen by cancer cells, and eventually exploit it therapeutically. The evolution of Warburg's idea is shown in Fig. 1. Year by year, emerging hypotheses focussing on different aspects of the Warburg effect constitute an increasingly complex puzzle, providing the insights into landscape of cancer cell metabolism from different perspectives, both as far as mechanisms and benefits are concerned. The most obvious concept indicates the need to support intensive cell divisions and shift biosynthetic precursors into anabolic reactions branching from the glycolytic pathway. This would increase the rate of nucleotide, protein, and lipid synthesis, macromolecules that are essential for intensive cell growth and proliferation [9]. The other theories postulate the existence of a glucose-deficient tumor microcosm, which accounts for the competition of the tumor cells with the T cells for nutrients and in turn explains the rapid rate of glucose uptake, in parallel resulting with immunosuppression [10]. Emphasis was also laid on the necessity of a readily available source of regenerated NAD^+^, which is not only necessary for the continuity of glycolysis but also required as a coenzyme for oxidized biomass synthesis [11]. Another explanation of Warburg-type metabolic alteration is the “solvent capacity limitation” theory. It states that the physical volume of the cell is insufficient to accommodate an adequately large number of mitochondria that are required to meet the enormous energy demands of the tumor [12, 13]. According to it, the citric acid cycle (TCA) is a key metabolic center responsible for supporting tumor growth [14, 15]. The “metabolic plasticity” theory allows precise switching between efficient phosphorylation and glycolysis, which is an adaptation of the tumor cell to live in different microenvironments, thereby questioning Warburg hypothesis of mitochondrial damage [16]. Consistent with the above, there is clear evidence that glycolysis is elevated in most tumors without mitochondrial dysfunction. Intensively proliferating tumor cells have an increased demand for ATP (Adenosine triphosphate) to meet the demand for metabolic reactions related to growth and cellular needs for ATP-dependent membrane pumps, e.g., Na^+^/K^+^ ATPase pump. Recent studies have correspondingly reported that changes in the cellular environment entail intensive aerobic glycolysis at a constant rate of oxidative phosphorylation to meet this demand. This provides metabolic flexibility in a high-energy demand situation for the cell [17]. On the other hand, some studies have proven that the Warburg effect can just as well be caused by mutation of the mitochondrial genes coding fumarate hydratase, succinate dehydrogenase, and isocitrate dehydrogenase necessary for TCA to take place, as well as overproduction of reactive oxygen species (ROS) by mitochondria [8, 18].

### Involvement of tumor niche

In the context of increased ROS formation, the simplistic Warburg effect model evolved into the so-called reverse Warburg effect [19]. It assumes close metabolic cooperation between activated fibroblasts of the stroma and the tumor cells, demonstrating that tumor cells mainly respire aerobically, using the lactate obtained from aerobic glycolysis occurring in tumor stromal fibroblasts [19]. The ROS released from the tumor cells reciprocally induces oxidative stress in the stromal fibroblasts. It leads to HIF-1α (hypoxia-inducible factor 1-alpha) activation and further glycolysis enhancement in the stromal fibroblasts. Thus, through oxidative stress and numerous catabolic processes such as autophagy, mitophagy, and fermentation (the process of conversion of a single molecule of glucose into two lactate molecules), aerobic glycolysis leads to the formation of lactate, an oncometabolite providing a favourable environment for tumor growth and proliferation [16]. On the other hand, rapid cell division results in hypoxia in a tumor microenvironment, where oxygen demand exceeds its supply. Additionally, the presence of hypoxic niches correlates with abnormal blood vasculature, the formation and collapse of which result in hypoxia/re-oxygenation cycles. Consequently, the tumor center is relatively more hypoxic and predominantly glycolysis dependent, while the more vascularized tumor periphery relies on mitochondrial respiration in accordance with the “metabolic plasticity” theory. Moreover, these two dissimilar tumor cell populations may be metabolically linked, and substrates from different cancer cell populations may be shared and utilized. These dynamic changes of hypoxia/reoxygenation cycles induce oxidative stress, and the resulting ROS may induce the previously mentioned changes in CAFs (cancer-associated fibroblasts), leading to their metabolic reprogramming towards aerobic glycolysis [20–22].

## Mechanism of Warburg effect

### Signal transduction pathways

Undoubtedly, Otto Warburg was the first to demonstrate that one of the characteristic features of cancer cells is the utilization of the aerobic glycolysis pathway for glucose metabolism [3]. The Warburg effect is inherently associated with extensive glucose uptake and metabolism, which the cancer cell achieves through metabolic reprogramming. Metabolic reprogramming is primarily the consequence of aberrant expression of transcription factors such as HIF-1, c-MYC (cellular myelocytomatosis oncogene), p53 (protein,P53) and the PI3K/Akt/mTOR (phosphoinositide 3-kinase/protein kinase B/mammalian target of rapamycin) signaling pathways [8, 23, 24]. mTOR is a serine/threonine kinase activated by various oncogenic signaling pathways and thus is overactive in cancer cells. mTOR is present as two multiprotein complexes, mTORC1 (mechanistic target of rapamycin complex 1) and m,TORC2, and is regulated by the PI3K/Akt signaling pathway, which integrates growth factor signaling with tumor cell metabolism, as well as the LKB1/AMPK (liver Kinase B1/5′AMP-activated protein kinase) pathway, controlling the energy status of the cell [25, 26]. mTOR amplifies the Warburg effect by stimulating the normoxic upregulation of the HIF-1 transcription factor among others.

### HIF-1—c-Myc interplay in normoxia

HIF-1 is a heterodimer composed of a constitutively expressed beta subunit and an oxygen-dependent alpha subunit. Under conditions of reduced partial pressure of oxygen, the alpha subunit is stabilized and the HIF-1 factor as a heterodimer is translocated to the cell nucleus, where it stimulates the expression of genes coding for glycolytic enzymes and glucose transporters [27]. Among downstream mTOR effectors, is the transcription factor c-MYC. In normal cells, c-MYC is inhibited by HIF-1, but in cancer cells it interacts with HIF-1, further enhancing the Warburg effect by increasing the expression of glycolytic enzymes [28]. However, the most notable mechanism is the stimulation of the Warburg effect by mTOR/HIF-1/MYC through the upregulation of PKM2 (pyruvate Kinase M2) expression [29]. It has been shown that genetic manipulations enabling the switch of expression from PKM2 to PKM1 can effectively reverse the Warburg effect. Therefore, it seems that PKM2 expression is crucial for promoting aerobic glycolysis [30]. PKM2 is a protein present in embryonic cells, normal proliferating cells, and cancer cells. PKM2 stimulates tumor growth through dual function, acting as a glycolytic enzyme in the cytosol and as a protein kinase in the cell nucleus, leading to enhanced expression of many proteins. In cancer cells, PKM2 is present in the cytosol in the form of a dimer with low catalytic activity, which allows it to direct glycolytic intermediates on the path of anabolic transformations [31]. In addition, PKM2 interacts with the alpha subunit of HIF-1 to stimulate further metabolic reprogramming [32]. Moreover, studies have shown that pyruvate and to a lesser extent, lactate, also lead to the stabilization of the alpha subunit of HIF-1, implying the existence of a positive feedback loop [33, 34]. Thus, from the metabolic viewpoint, intensive glycolysis will lead, via the transcription factor HIF-1, to the increased expression of glycolytic enzymes and glucose transporters. Additionally, the HIF-1 factor, by upregulating the expression of PDK1 (pyruvate dehydrogenase kinase isoform 1), stimulates the phosphorylation of pyruvate dehydrogenase, and thus reduces its activity, further enhancing the Warburg effect [35].

### Environmental selection of Warburg phenotype cells

Undoubtedly, normoxic stabilization of the HIF-1 transcription factor constitutes a single most crucial mechanism behind the metabolic reprogramming (Warburg effect) in cancer cells. However, it should be noted that hypoxia itself may also lead to the selection of cells with the Warburg phenotype (cells undergoing aerobic glycolysis). Recent studies have shown that conditions of low glucose, low oxygen, and low pH or starvation constitute a strong selective environment for acquiring the Warburg phenotype by cancer cells, as a result of changes at the level o,f the genome, transcriptome, and epigenome [36]. On the other hand, selection in a hypoxic environment does not seem to constitute a universal/mandatory mechanism for Warburg phenotype acquisition as another study has shown that cells selected under hypoxic conditions generated more energy via mitochondrial respiration than control cells [37].

### Loss of p53 drives the Warburg effect

Another piece in the puzzle is the p53 (a crucial tumor suppressor) protein, as loss of its function also plays a role in the induction of the Warburg effect. The p53 protein regulates glucose metabolism by inhibiting the expression of glucose transporters and the activity of PFK-1 (phosphofructokinase-1). The product of the TIGAR gene, controlled by p53, reduces the activity of PFK-1 by reducing the availability of its allosteric activator fructose 2,6-bisphosphate (F-2,6-P2) [38]. The p53 protein also inhibits glycolysis indirectly by increasing the expression of PTEN, an inhibitor of the PI3K/Akt pathway [35]. Thus, releasing glycolysis from the inhibitory control of the p53 protein eventually contributes to enhancing the Warburg effect.

## Warburg effect—advantages for cancer cells

### Rapid ATP synthesis and NAD^+^ regeneration

Studies have shown that glucose and glutamine are the compounds most intensively metabolized in many cancers [39, 40]. The transformation of these molecules provides, for the most part, adequate amounts of nitrogen and carbon skeletons, as well as reducing potential and free energy necessary to maintain cellular growth. Undoubtedly, the crucial benefit of the Warburg effect is an increased ATP production rate as aerobic glycolysis can generate ATP faster than the rate of oxidative phosphorylation [41]. Intensive production of lactate, on the one hand, determines the rapid recovery of NAD^+^ (via lactate dehydrogenase, converting pyruvate to lactate), which allows for maintaining a high rate of glucose consumption and an appropriate level of ATP for rapid proliferation. However, on the other hand, it should be noted that the relationship between the NADH formed during glycolysis and the NAD^+^ obtained during lactate production by lactate dehydrogenase remains equal. Therefore, if the oxidized intermediates of glycolysis are directed into other metabolic pathways, e.g. the glycine-serine-nucleotide axis, starting from oxidation of 3-phosphoglycerate to 3-phosphohydroxypyruvate, the substrate of subsequent transamination, the NADH formed in the oxidation step must be regenerated by another red/ox reaction, for example using respiratory chain upon reducing equivalent shuttling from cytoplasm into mirochondria [42] or additional source of pyruvate is used, e.g. from the metabolism of glutamine [43]. Undoubtedly, the recently published work of Vander Heiden’s group shed new light on this issue. Their research shows that the utilization of NAD^+^ in cancer cells may be faster than the usage of ATP. As a consequence, the energy stored in the mitochondrial proton gradient is harnessed by ATP synthase to phosphorylate ADP (adenosine diphosphate) to make ATP. The results obtained also suggest that the higher demand for NAD^+^ about the demand for ATP leads to a reduction in mitochondrial respiration and thus a decrease in the NAD^+^/NADH ratio, promoting fermentation even when oxygen is available. Indeed, inducing ATP hydrolysis in the cell, which provides ADP for ATP synthase, released NAD^+^ regeneration by ETC (electron transport chain) as well as proliferation with diminished dependency on fermentation [11].

By what is mentioned above, tumors have a high demand for NAD^+^ to synthesize biomass, thus cancer cells often have high expression of nicotinamide phosphoribosyltransferase (NAMPT), the rate-limiting enzyme, converting nicotinamide (NAM) to nicotinamide mononucleotide (NMN), which is then converted to NAD^+^. This pathway is crucial to cancer cells, as it converts NAM, the catabolic product of NAD^+^-consuming enzymes, back to NAD^+^ [44, 45]. In conclusion, studies by Luengo et al. have shown that the maintenance of the optimal NAD^+^ pool is a key condition for maintaining a high proliferative potential, and thus the growth of cancer cells [11]. Of note, regardless of the source of lactate in the tumor cell, its formation not only enables NADH to be oxidized to NAD^+^ but lactate itself acts as an oncometabolite influencing the tumor microenvironment several of mechanisms that will be discussed later in the article.

### Biomass and NADPH production

**Fig. 2:**
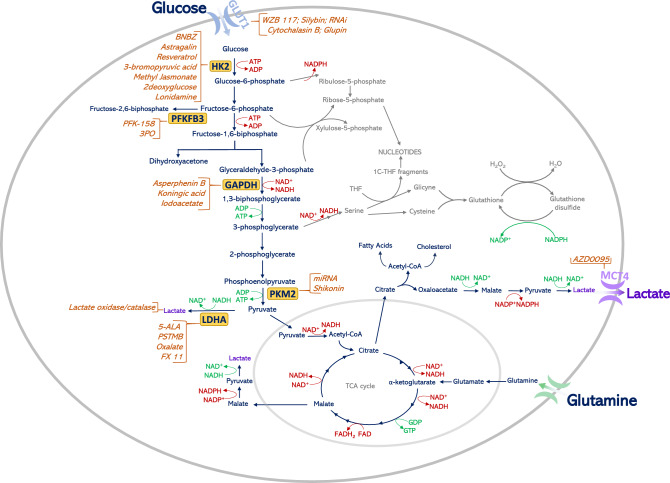
Warburg effect advantage for cancer cells—biomass and NADPH production. The main metabolic pathways contributing to biomass production in a cancer cell as nucleotide synthesis, the pentose phosphate pathway, serine synthesis, glutaminolysis, cholesterol synthesis, and fatty-acid synthesis are presented. In addition, the inhibitors of enzymes/proteins involved in the Warburg effect together with their targets are presented on the graph. *ADP* adenosine diphosphate, *ATP* adenosine triphosphate, *GAPDH* glyceraldehyde 3-phosphate dehydrogenase, *GLUT-1* glucose transporter 1, *HK-2* hexokinase-2, *LDHA* lactate dehydrogenase A, *MCT-4* monocarboxylate transporter 4, *NAD+* nicotinamide adenine dinucleotide, oxidized form, *NADH* nicotinamide adenine dinucleotide, reduced form, *PFKFB3* 6-phosphofructo-2-kinase/fructose-2,6-biphosphatase 3, *PKM2* pyruvate kinase M2, *TCA* tricarboxylic acid, *THF* tetrahydrofolate

Rapid glucose metabolism besides providing energy, supplies precursors for anabolic processes, including reducing equivalents in the form of NADPH (nicotinamide adenine dinucleotide phosphate) (Fig. 2). Intermediates for the synthesis of nucleotides and non-essential amino acids are also furnished by such high glucose turnover. An example is the usage of a glycolysis intermediate, 3-phosphoglycerate, for the synthesis of serine, which in successive transformations is a donor of a one-carbon fragment for nucleotide synthesis, while at the same time being a precursor for glycine synthesis. Thus, under conditions of limited availability of serine, its de novo synthesis from the glycolysis intermediate ensures that the tumor cell maintains its proliferative potential. Another example is the use of glyceraldehyde-3-phosphate and fructose-6-phosphate, intermediates of glycolysis, in the synthesis of ribose-5-phosphate (R-5-P) via the non-oxidative arm of the pentose phosphate pathway (PPP). It should also be noted that of all the glucose that enters the cancer cell, some does not enter the glycolysis pathway at all, and is directly metabolized in the PPP oxidative arm, which is an alternative to the aforementioned R-5-P synthesis route and is additionally a source of NADPH. NADPH provides the reducing power required for the synthesis of fatty acids, sterols, nucleotides, and non-essential amino acids. Of note, if a cell requires more NADPH than a precursor for nucleotide synthesis, the excess ribulose 5-phosphate is then converted to compounds entering glycolysis in a series of reversible reactions of PPP non-oxidative arm. Thus, directing glucose into the oxidative arm of PPP does not exclude its subsequent transformation to lactate [46–48]. Maintaining the appropriate level of NADPH is also crucial to keep the appropriate level of reduced glutathione, protecting the cell against ROS leading to free-radical damage [46]. The concentration of ROS in cancer cells is usually high, and this promotes DNA damage and cancer progression, simultaneously having a cascading toxic effect on all cellular structures [49]. As has been shown by Anastasiou et al., increased ROS formation inhibits PKM2 isoenzyme through cysteine oxidation leading to higher glucose flux into the PPP, which in consequence decreases the oxidative stress by increasing the level of reduced glutathione [50]. Other important proteins which can be involved in glucose flow between glycolysis and the oxidative arm of PPP are tumor-specific isoenzymes of phosphofructokinase II, PFKFB3 (6-phosphofructo-2-kinase/fructose-2,6-biphosphatase 3) and PFKFB4. These proteins are highly expressed in cancer cells. It was shown that PFKFB3 acts mainly as a kinase that will promote glycolysis by stimulating the synthesis of F-2,6-BP (fructose-2,6-bisphosphate), while PFKFB4 may occur in two forms, kinase, and phosphatase. As a phosphatase, PFKFB4 hydrolyses F-2,6-BP, the glycolysis activator, and in consequence, more glucose can be directed into an alternative PPP pathway [51].

### Lactate as an oncometabolite

The increased production of lactate associated with the Warburg effect results in a decreased pH and thus provides an acidic tumor microenvironment (TME). The lactate generated together with H+ ions, which are formed mainly in the process of converting glucose into pyruvate, are transported through the cell membrane with the help of MCT1 (monocarboxylate transporter 1) and MCT4 (monocarboxylate transporter 4) transporters, on a symport basis, a form of secondary active transport [52]. Moreover, a change in the pH in the extracellular microenvironment of the tumor stimulates its development, promoting cell spreading, and consequently leads to metastasis [53]. Tumor progression is manifested in three aspects: (1) extracellular matrix (ECM) degradation and tumor cell migration (2) angiogenesis and (3) immunosuppression [54]. In each of these aspects, lactate plays an indirect role, providing a low pH environment, a factor that directly affects tumor progression. The process of direct migration of tumor cells begins with ECM degradation. Low pH activates enzymes such as matrix metalloproteinases (MMPs) released from the tumor cell, causing digestion of the surrounding matrix—this allows the cells to detach from the solid substrate. A direct correlation between the concentration of lactate and the activity of proteinases was demonstrated for cathepsin B, hyaluronidase-2 [55], and matrix metalloproteinases-9 [56]. The next step is the migration (via lymphatic/ hematogenous/direct or trans-coelomic routes) and attachment of free cancer cells to other tissues. Due to lactic acidosis, the production of actin filaments is activated [57], the properties of integrins on the surface of cancer cells [58] are changed, the number and size of 'invadopodia' (adhesive, membrane structures, containing proteases responsible for ECM degradation) are increased [59] and the expression of hyaluronan and CD44 (cluster of differentiation 44) is increased [60]. These processes allow for the movement and adhesion of free cancer cells to healthy tissues, and at a later stage, for the development of metastases. Studies have confirmed a positive correlation between high lactate levels and metastases in cervical cancer [61], head and neck cancers [62], colorectal adenocarcinoma [56, 63] and lung cancer [64]. Moreover, lactate is suggested also as an important player in the pathogenesis of metastatic spine disease [54] and generally high lactate concentrations in cancer cells have been associated with an overall more aggressive disease course and reduced chances of survival [65]. High lactate concentration in tumor cells affects also the process of angiogenesis through two distinct pathways, depending on the individual products of the lactate dehydrogenase (LDH) reaction. In the first case, the accumulation of pyruvate leads to the inhibition of prolyl hydroxylases (PHDs), resulting in a decreased degradation of HIF-1α [66]. The HIF-1ɑ factor activates the expression of proangiogenic mediators such as bFGF (basic fibroblast growth factor), SDF-1 (Stromal cell-derived factor 1), and VEGF (Vascular endothelial growth factor) [67]. In the second case, the accumulation of pyruvate leads to the accumulation of NADH, which activates the NADPH oxidase enzyme and promotes the formation of superoxide anion radical, resulting in a cascade of subsequent effects: it leads to ROS-dependent degradation of IĸBɑ (NF-κB protein inhibitor), activation of NF-kB (Nuclear factor kappa B) and the expression of IL-8 (interleukin 8)—a pro-angiogenic cytokine [64, 68]. Lactate also impairs the immune response in the tumor microenvironment, through a variety of effects on the cells of the immune system: T-cells, natural killers (NK) and natural killer T cells (NKT), dendritic cells, and macrophages [69]. In the case of T-cells, a high concentration of lactate has been linked to the inhibition of migration and cytotoxicity for CD4^+^ (cluster of differentiation 4) and CD8^+^ (cluster of differentiation 8) cells by disrupting lactate export [69], while a decrease in NAD^+^ T-cells’ levels, resulting from the presence of lactate, induces their apoptosis by suppressing FIP200 (focal adhesion kinase family interacting protein of 200 kD) [70]. Lactate blocks the production of pro-inflammatory cytokines: IL-4 and IFN by NKT cells, by inhibiting the mTOR signaling pathway [71]. A high lactate-to-glucose ratio stimulates lactate-avid Treg cells, which promote immunosuppression in the tumor environment [72]. Accumulation of lactate and the associated low pH in the tumor cell environment directly inactivates NK cells, causing their apoptosis and disrupting the regulation of the nuclear factor of activated T cells (NFAT), reducing the production of IFN-y (Interferon y). Dendritic cells (DCs), responsible for antigen presentation, increase the production of the immunosuppressive cytokine IL-10 (interleukin 10) as a result of lactate accumulation and limit differentiation [73]. In the presence of lactate, the anti-tumor function of M1 macrophages is inhibited by reducing the expression of IL-6 (interleukin 6), IL–1 (interleukin 1), iNOS (inducible nitric oxide synthase), and TNF (tumor necrosis factor) [72, 74], while inactivated macrophages undergo an induced polarization process changing their phenotypes, that results in the formation of pro-tumor M2 macrophages [75, 76]. The relationship between the presence of lactate in TME and immunosuppression has been demonstrated in the following cancers: breast cancer [77], prostate carcinoma [78], cervical cancer [79], and pancreatic cancer [80]. In conclusion, lactate plays an important role as an oncometabolite in the development of cancer, generating a low pH in the tumor microenvironment and participating in metabolic symbiosis. It is responsible for the greater malignant potential of tumor cells, and therapeutic resistance, and stimulates the loss of adhesion and subsequent migration of cancer cells leading to metastasis, angiogenesis, and immunosuppression in the vicinity of the tumor. Treatments based on the modification of the tumor microenvironment are the basis for potential future anti-cancer therapies [81].

## Reverse Warburg effect

Several ambiguities related to the functioning of tumor cells and, above all, the determination of scientists to solve the intricate metabolic puzzle, allowed Warburg's idea to evolve, through many different models and hypotheses, until 2009, when the so-called reverse Warburg effect was introduced into the scientific discourse [19]. According to the reverse Warburg effect hypothesis, lactate is the most important metabolic fuel for cancer cells because, through a metabolic symbiosis between cancer cells and cancer-associated fibroblasts, it enables tumor self-sufficiency for cancer cells [82].To delve into the essence of this mechanism, it's worth taking a close look at the cellular environment of cancer. All tumors have two basic components: neoplastic parenchymal cells and the associated stroma consisting of blood vessels, immune cells, and supporting cells. Their classification and biological behaviour are mainly decided by the parenchyma, whereas their growth and spread are determined by the stromal components. This microenvironmental stroma is formed by tumor-associated inflammatory cells, fibroblasts, lymphoid cells, and vascular endothelial cells. The reverse Warburg effect mechanism involves both activated fibroblasts and the parenchymal tumor cells. The differentiation of stromal cells into CAFs takes place in the extracellular compartment of the tumor under the influence of tumor cells [19]. The tumor cells disturb the physiological stroma and turn it into a factory of energy-rich metabolites [19]. Tumor cells also lead to oxidative stress on the fibroblasts by generating ROS in the form of H_2_O_2_ [83], resulting in numerous catabolic processes in the cells, such as autophagy, mitophagy, and lactic acid fermentation [84, 85].

**Fig. 3:**
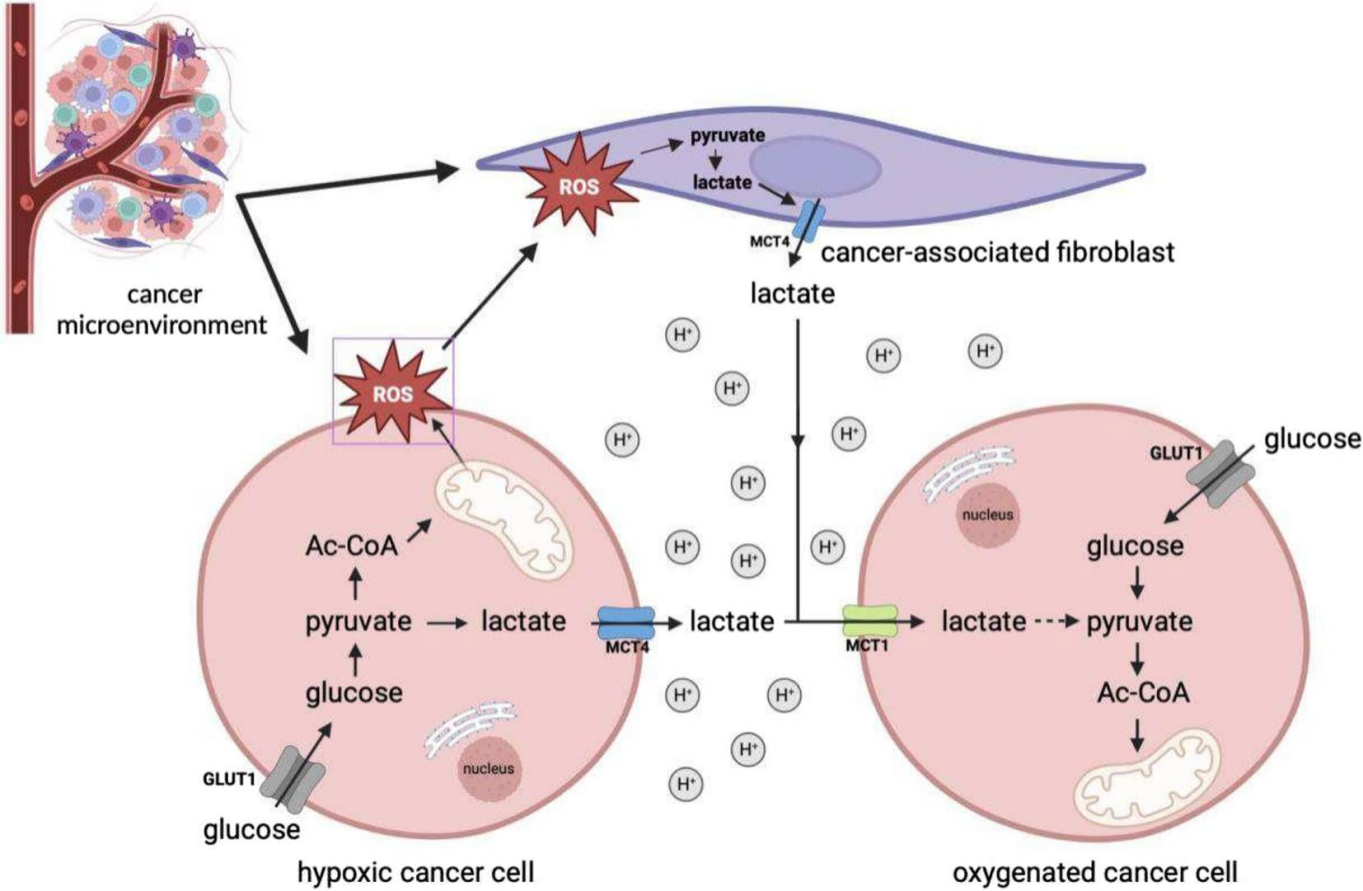
Reverse Warburg effect. In the reverse Warburg effect, oxidative cancer cells can take up lactate from hypoxic cancer cells. Moreover, oxidative cancer cells induce oxidative stress in cancer-associated fibroblasts (CAFs) by secreting reactive oxygen spiecies (ROS), which in turn triggers the aerobic glycolysis in CAFs. In consequence, lactate and pyruvate produced by CAFs are metabolized in adjacent oxidative cancer cells. *AC-CoA* acetyl coenzyme A

It is here that the role of a factory of energy-rich metabolites is revealed, as through these processes, CAFs provide cancer cells with large amounts of lactate, ketones, or glutamine, which are fuel for bio-synthetic reactions, numerous anabolic processes, and the production of large amounts of ATP [86, 87]. Thus, it would be correct to view the reverse Warburg effect as a parasitic metabolic pathway in which cancer cells have their metabolic source in the CAFs while generating ROS and creating conditions for the production of lactate by other cells. The pressure that tumors exert on fibroblasts gives them the phenotype of the Warburg effect proper—fibroblasts are the site of intensive aerobic glycolysis, while tumor cells generally respire normally and draw only pyruvate and lactate from CAFs. Therefore, the reverse Warburg effect is a two-compartment model showing metabolic symbiosis or metabolic coupling between CAFs and tumor cells. This mechanism is thus an alternative to the “ancestral mutation model,” as epithelial tumor cells instruct stromal cells to transform into the energy-rich stroma, thereby facilitating tumor growth and angiogenesis [19, 82, 88]. Glucose is the most important metabolite used by the neoplastic cells for ATP generation, production of essential cellular components and regulation of the redox state of cells [9]. Its catabolism, or aerobic glycolysis, is predominantly in the CAFs/ stromal fibroblasts that generate large quantities of lactate through it [82]. Lactate is consequently secreted from the cytoplasm of CAFs into the extracellular space. Normally, this entails the expression of the monocarboxyl transporters, MCT4 and MCT1 [88]. Most studies indicate that MCT4 has the lowest affinity for lactate (high Km) among MCTs and facilitates lactic acid efflux from glycolytic cells, including hypoxic cancer cells. MCT1, on the other hand, has a high affinity for lactate (low Km) and primarily mediates lactic acid influx, used subsequently as an oxidative fuel for mitochondrial respiration [89]. Of note, MCT4, as opposed to MCT1, is up-regulated by hypoxia, through a HIF-1- dependent mechanism, promoting lactate efflux from hypoxic cells [90]. High expression of both, MCT1 and MCT4, is observed in many tumors which usually correlates with poorer overall survival [89]. However, in triple-negative breast cancer and in non-small cell lung cancer, high expression of only MCT4, not MCT1, correlated with a worse prognosis [91, 92]. Understandably, these transporters are overexpressed also in CAFs [93]. Accumulated lactate, other secretions from tumor cells and oxidative stress cumulatively create conditions, which differentiate tumor cells into two major subsets: hypoxic tumor cells and oxidative tumor cells, and the cause of their intrinsic metabolic heterogeneity [82, 94]. Hypoxic tumor cells direct their metabolism toward anaerobic glycolysis, generating lactate, as in CAFs [93, 95]. The metabolites mainly lactate and small amounts of pyruvate are then exported from the cytoplasm of the cells to the extracellular space by the previously mentioned specific MCT4 transporters [19]. Thus, the lactate generated by these cells and CAFs provides a huge and ready pool of fuel for neighboring tumor cells to meet their metabolic demands [96]. Its transport into the interior of these cells is possible via MCT-1 transporters, which are the main importers of lactate [97, 98]. Such metabolic flexibility owes its existence to an efficient lactate shuttle (Fig. 3). Lactate that is not utilized in this way is deposited in the extra-cellular space and results in the formation of an acidic microenvironment, a condition that further promotes carcinogenesis [52]. Unused lactate is metabolized by oxidative phosphorylation, resulting in the generation of ATP in normoxic tumor cells. Importantly, the vast majority of cells in the tumor microenvironment are oxidative cells, which, according to the presumptions of the reverse Warburg effect, generate the greatest amount of energy in the form of ATP, which is essential for tumor growth, proliferation, progression, and most importantly, metastasis. To achieve it, they use a bidirectional enzyme—lactate dehydrogenase responsible for the conversion of lactate back to pyruvate, which enables its subsequent incorporation into the Krebs cycle and ultimate energy acquisition via the oxidative pathway [99]. It is worth noting that LDH consists of two subunits (M) and (H) codified by the LDHA and LDHB genes, respectively. These subunits can form 5 different combinations of homo and heterodimers LDH-1 (4H), LDH-2 (3H1M), LDH-3 (2H2M), LDH-4 (1H3M) and LDH-5 (4 M). In cancer cells, LDH-1 and LDH-5 are preferentially expressed. The LDH-5, coded by the *LDHA* gene preferentially converts pyruvate to lactate, whereas the LDH-1, coded by the *LDHB* gene, opposite, lactate into pyruvate. Thus, lactate-forming cells will have higher expression of LDHA, whereas lactate-consuming cells will preferentially express the *LDHB* gene [100]. As high expression of *LDHA* and/or *LDHB* is associated with poor prognosis, these enzymes, as well as MCT1 and MCT4 lactate transporters, can serve as important therapeutic targets for anti-cancer therapy. Interestingly, a recent study, analyzing metabolic signature in human cancers at the single-cell level, revealed the coexistence of glycolytic and mitochondrial metabolic signature among cells in the same tumor, confirming “metabolic plasticity” and indicating the reverse Warburg effect between different cancer cell subpopulations [101]. Thus, targeting a protein/proteins associated with different metabolic profiles seems to be an optimal approach for an anti-cancer strategy.

## Warburg effect—as a potential therapeutic target

Given the multifaceted nature of the Warburg effect—both in terms of causes and benefits for the transformed cell, it seems an obvious target in new therapeutic regimens. However, the question arises—which dominoes to hit to “collapse” the cancer? Recent years have shown that the Warburg effect may not only be about the rapid generation of ATP and directing intermediates to biosynthetic pathways, but it may also result from the too-slow recovery of NAD^+^ in relation to the rate of energy generation in the form of ATP. In such a situation, it is cost-effective for the cell to regenerate NAD^+^ by promoting fermentation. Another issue is the reverse Warburg effect, which itself could become a therapeutic target as well. The very initiation of the reverse Warburg effect is a consequence of the production of ROS, so reducing the oxidative stress in cancer cells could lead to the inhibition of the reverse Warburg effect. Thereby, another domino cubes come here, the enzymes PFKFB3 and PFKFB4, which direct the flow of glucose between the PPP pathway and glycolysis. However, to become an important therapeutic target, the catalytic function of these enzymes would have to be well recognized in particular cancer. According to the latest data, it is the expression at the level of these isoforms to determine whether phosphatase activity will dominate [102], which directs glucose to PPP increasing the same protection against ROS, or kinase activity, accelerating the glycolysis pathway. Another issue is lactate acting as an oncometabolite mediating migration, invasion, immunosuppression, and angiogenesis. It would seem that the inhibition of LDH will not only inhibit the regeneration of NAD^+^, but also the production of this significant cancer metabolite, and will prevent the use of lactate as an energy fuel in the reverse Warburg effect. On the other hand, many normal cells of the immune system rely on aerobic glycolysis, and such intervention would still promote tumor-promoting immunosuppression. A more effective and tumor-selective intervention in the reprogrammed metabolism of cancer cells would be the use of the lactate-consuming enzyme; lactate oxidase, an approach that, especially in the form of targeted nanoparticles, should bring more benefits and fewer side effects [103]. Table 1 presents inhibitors of selected proteins involved in the Warburg effect, currently under investigation for treating cancer. Some of these compounds entered the clinical phase of trials, showing an inhibitory effect on tumor growth, as revealed by recent studies. Despite some issues concerning drug delivery and bioavailability, specificity, and toxicity, cancer cell plasticity, and heterogeneity of tumor cells that make it difficult to apply monotherapy schemes, glucose metabolism inhibitors still have anticancer potential as components of multi-agent regimens [24, 104]. The reader can find much more detailed information in the latest publications devoted to the inhibition of anaerobic glycolysis in the context of improving some of the currently used anticancer therapies. Numerous basic preclinical research has focused on multiple Warburg effect inhibitors, as single agents or in combinations, but few of them have entered clinical trials [104, 105]. Among them there are 2-deoxy glucose (hexokinase inhibitor, tested alone or in combination with docetaxel in patients with advanced solid tumors) [106], lonidamine (hexokinase inhibitor, tested in combination with various chemotherapeutic agents, e.g. 5-fluorouracil, doxorubicin, cisplatin in several advanced cancer) [107], indisulam (carbonic anhydrase inhibitor, tested in combination with capecitabine/Irinotecan in metastatic colorectal cancer) [108] and AZD3965 (MCT1 inhibitor, tested alone in some lymphomas and solid tumors) [104]. To date, none of the tested compounds, alone or in combination, have brought satisfactory results allowing for use in routine clinical practice. Nevertheless, one should be aware that in the era of constantly developing theranostic and increasingly advanced research possibilities, it is more than likely that the general findings made on cancer models (cell lines, animals, groups of selected cancer patients) may not be applicable to large cohorts of patients, but could evince great importance in the individualized approach to individual patients.

**Table 1:** Inhibitors of selected proteins involved in the Warburg effect

Target	Agent	State of development
GLUT-1	WZB 117	In vivo [109]
	Cytochalasin B	In vivo [110]
	Glupin	In vitro [111]
	Silybin	Clinical phase II [112]
	RNAi	Early clinical phase I [113]
Hexokinase	2-Deoxyglucose	Clinical phase II [114]
	Lonidamine [115]	Clinical phase III [116]
	3-Bromopyruvic acid [117]	Clinical phase I [118]
	Methyl Jasmonate	In vitro [119]
	BNBZ	In vivo [120]
	Astragalin	In vitro and in vivo [121]
	Resveratrol [122]	Clinical phase II [123]
Glyceraldehyde-3-phosphate dehydrogenase (GAPDH)	Asperphenin B	In vivo and in vitro [124]
	Koningic acid	In vitro [125]
	Iodoacetate	In vitro [126] and in vivo[127]
Lactate dehydrogenase A (LDHA)	5-ALA	Clinical phase II study record | Beta ClinicalTrials.gov. https://beta.clinicaltrials.gov/study/NCT05101798
	Oxalate	In vivo [128]
	FX 11	In vitro [129, 130]
	PSTMB	In vitro [131]
Pyruvate kinase isoform M2 (PKM2)	miRNA	In vitro [132]
	Shikonin	Clinical phase I [133]
PFKFB3	PFK-158	Clinical phase I [134]
	3PO	Preclinical phase [134]
MCT1	AZD3965	Clinical phase I [135]
MCT4	AZD0095	Preclinical phase [136]
Lactate	Lactate oxidase/catalase	Preclinical phase [103]

*BNBZ* benitrobenrazide, *PSTMB* 1-(phenylseleno)-4-(trifluoromethyl) benzene, *5-ALA* 5-aminolevulinic acid, *3PO* 3*-*(3-pyridinyl)-1-(4-pyridinyl)-2-propen-1-one, *RNAi* RNA interference, *miRNA* microRNA

## Concluding remarks

Despite researchers’ efforts, cancer cell metabolism still eludes a full understanding and clarification of the complex interconnections in terms of intracellular, cancer cell-cancer cell, and cancer cell-tumor stroma interactions. The seemingly simple metabolic reprogramming observed in transformed cells by Otto Warburg about a century ago, later recognized as one of the main hallmarks of cancer, is even now an area of detailed investigation, gradually uncovering the complexity of tumor functioning. Behind this effect of increased “aerobic glycolysis”, leading to lactate production despite normal oxygen supply, is a dysregulated intracellular signaling, enhanced biomass production potential, efficient energy supply as well as the increased metastatic ability or immunosuppression. In concert with intratumor interrelationship, where populations with different metabolic activities (based on glycolysis or oxidative phosphorylation) coexist, cancer cells take advantage of cancer-associated fibroblasts by first forcing their metabolic reprogramming and then consuming its products. In view of the presented facts and hypotheses regarding metabolic reprogramming in cancer cells, it is more than obvious that the more closely this phenomenon is studied, the more metabolic connections emerge. The issue seems even more intricate as what was previously considered the main benefit of the Warburg effect for cancer cells may no longer be so in light of recent research. Therefore, only a better understanding of the mechanisms of metabolic alterations and their interrelations can allow for an accurate and effective hit in the changed metabolism, which undoubtedly determines the rapid proliferation and tumor biomass production. Clearly, targeting specific nodal points regulating metabolite flux in tumors rather than targeting individual glycolytic enzymes should bring the desired effects in terms of specificity and treatment efficacy.

## Data Availability

The submitted manuscript has no associated data.

## Abbreviations

ADP: Adenosine diphosphate
Akt: Protein kinase B
AMPK: 5′AMP-activated protein kinase
ATP: Adenosine triphosphate
BFGF: Basic fibroblast growth factor
CAFs: Cancer-associated fibroblasts
CD4+: Cluster of differentiation 4
CD44: Cluster of differentiation 44
CD8+: Cluster of differentiation 8
c-Myc: Cellular myelocytomatosis oncogene
DCs: Dendritic cells
ECM: Extracellular matrix
ETC: Electron transport chain
F-2,6-BP: Fructose-2,6-bisphosphate
F-2,6-P2: Fructose 2,6-bisphosphate
FIP200: Focal adhesion kinase family interacting protein of 200 kD
GAPDH: Glyceraldehyde 3-phosphate dehydrogenase
GLUTs: Glucose transporters
HIF-1α: Hypoxia-inducible factor 1-alpha
IFN-γ: Interferon γ
IkBα: NF-κB protein inhibitor
IL-1: Interleukin 1
IL-10: Interleukin 10
IL-6: Interleukin 6
IL-8: Interleukin 8
iNOS: Inducible nitric oxide synthase
LDH: Lactate dehydrogenase
LKB1: Liver kinase B1
MCT1: Monocarboxylate transporter 1
MCT4: Monocarboxylate transporter 4
MMPs: Matrix metalloproteinases
mTOR: Mammalian target of rapamycin
mTORC1/2: Mechanistic target of rapamycin complex 1/2
NAD+: Nicotinamide adenine dinucleotide, oxidized form
NADH: Nicotinamide adenine dinucleotide, reduced form
NADPH: Nicotinamide adenine dinucleotide phosphate
NAM: Nicotinamide
NAMPT: Nicotinamide phosphoribosyltransferase
NFAT: Nuclear factor of activated T-cells
NF-kB: Nuclear factor kappa B
NKT: Natura killer T cells
NMN: Nicotinamide mononucleotide
p53: Protein P53
PDK1: Phosphoinositide-dependent kinase-1
PFK1: Phosphofructokinase-1
PFKFB3/4: 6-Phosphofructo-2-kinase/fructose-2,6-biphosphatase 3/4
PHDs: Prolyl hydroxylases
PI3K: Phosphoinositide 3-kinase
PKM2/1: Pyruvate kinase M2/M1
PPP: Pentose phosphate pathway
R-5-P: Ribose-5-phosphate
ROS: Reactive oxygen species
SDF-1: Stromal cell-derived factor 1
TME: Tumor microenvironment
TNF: Tumor necrosis factor
VEGF: Vascular endothelial growth factor
